# A novel scoring system for evaluating mortality risk of patients with sepsis during early hospitalization

**DOI:** 10.1186/s12879-025-10920-8

**Published:** 2025-07-01

**Authors:** Ivan Aranza, Miro Vuković, Valentina Biloš, Alen Juginović

**Affiliations:** 1 Department of Cardiology, University Hospital Split, Spinčićeva 1, Split, 21000 Croatia; 2 Center for Evidence-Based Medicine, University of Split School of Medicine, Šoltanska 2A, Split, 21000 Croatia; 3 University of Split School of Medicine, Šoltanska 2A, 21000 Split, Croatia; 4 Department of Neurobiology, Harvard Medical School, 220 Longwood Avenue, Boston, MA 02115 USA

**Keywords:** Sepsis, Early hospitalization, Emergency department, Chlorides, Mean corpuscular hemoglobin, Red blood cell distribution width, Phosphates, pH, Partial thromboplastin time, Lactate dehydrogenase

## Abstract

**Background:**

Sepsis is a complex life-threatening condition. Early initiation of treatment is crucial in reducing mortality. Current scoring systems have a lack of reliability in the emergency department (ED) or during early hospitalization (EH). Thus, a quick, reliable, and objective scoring system for assessing mortality risk during EH or ED could significantly reduce sepsis mortality.

**Methods:**

Using the MIMIC-IV database, we identified 7546 patients hospitalized due to septicemia. We included 13 comorbidity groups and the first chronologically available values of 75 laboratory parameters from the ED or EH. To create and validate our scoring system for early prediction of in-hospital mortality (e-SEPSS), patients were assigned to model development (MD) (*N* = 1004) or model validation (MV) group (*N* = 6542), with the latter serving as internal validation of e-SEPSS. Each risk factor that contributed significantly to mortality was assigned one point. Groups with different numbers of points were compared according to mortality and hospitalization duration.

**Results:**

Decreased chlorides, increased mean corpuscular hemoglobin, increased red blood cell distribution width, increased phosphates, decreased pH, increased partial thromboplastin time, and increased lactate dehydrogenase were included in e-SEPSS due to the highest reliability in predicting mortality. Patients received 1 point for each parameter, creating 8 mortality risk groups. A significant linear increase in mortality with each additional point was shown, ranging from 4.1% (0 points) to 100% (7 points) in the MD group. Similar trends were observed in the MV group. High power in discriminating patients with different mortality risks was shown (MD (ROC AUC = 0.718, CI 0.682–0.754), MV (ROC AUC = 0.798, CI 0.783–0.813)). Decreased survival time and shorter time-to-death with each additional point strengthened the validity of e-SEPSS (Mantel-Cox χ^2^(7) = 994.2, p-value < 0.001).

**Conclusion:**

e-SEPSS provides a simple, objective, reliable, and accessible way of predicting mortality in septic patients in the ED or during EH.. After external and clinical validation of e-SEPSS, it could become a useful additional tool in reducing sepsis mortality.

**Supplementary Information:**

The online version contains supplementary material available at 10.1186/s12879-025-10920-8.

## Introduction

Sepsis is defined as life-threatening organ dysfunction caused by a dysregulated host response to infection [1]. Data from 2017 show that sepsis contributes to 1 in 3 hospital deaths, while also representing a huge economic burden to healthcare systems worldwide, particularly in low and middle-income countries, making it one of the major challenges of modern healthcare [2–7]. However, sepsis is a treatable condition, and timely fluid resuscitation with early antibiotic administration has been shown to reduce mortality by as much as 26%^2^, ^8^. Delayed initiation of treatment can result in septic shock with even greater risk of mortality [1]. Keeping in mind that patients with septic shock exhibit a 35% increased risk of mortality for every hour of delayed antibiotic treatment, it is clear that timely, efficient, and precise diagnosis of sepsis is a prerequisite for any successful treatment effort [9–12]. However, the multifactorial and complex etiopathogenesis of sepsis presents challenges for physicians in accurately triaging patients into groups by severity of disease at hospital admission, particularly due to the heterogeneous signs and symptoms exhibited by sepsis patients [13–15]. After triage, patients with sepsis are commonly treated in intensive care units (ICUs) where their prognosis is assessed using numerous general severity-of-illness scores, such as Acute Physiology and Chronic Health Evaluation (APACHE), Sequential Organ Failure Assessment (SOFA), and Simplified Acute Physiology Score (SAPS), which are calculated using a variety of clinical and laboratory parameters [16–19]. These scores have limitations such as time needed for calculation, varying clinical objectiveness, and lack of specificity for sepsis that preclude them from reliably predicting sepsis mortality in the early stages, ideally at admission, which could have significant negative consequences on patient outcomes [20–23].

Thus, there is a clear need for a rapid and reliable screening method that can be used at admission and during early hospitalization (EH) before the patients arrive in the ICU to reduce sepsis mortality since it would allow physicians and hospital staff to allocate resources more effectively and initiate prompt and adequate treatment. This screening method should be affordable, accessible to almost any healthcare facility worldwide, and simple, while maintaining statistically high relevance for predicting sepsis outcomes [24].

## Methods

### Study design

We conducted a retrospective cohort study using data from the MIMIC (Medical Information Mart for Intensive Care) database. MIMIC is a large and freely available database that consists of de-identified health-related data from patients admitted to the critical care units of Beth Israel Deaconess Medical Center in Boston, MA, USA [25, 26]. It has several versions, while the one used in this article, MIMIC-IV, contains data from 2008 to 2019 composed of several comprehensive data streams in the intensive care environment, with high levels of richness and detail. MIMIC-IV intends to support complex signal processing and clinical querying that could ultimately lead to improved patient outcomes. It was a coordinated endeavor of the academy, industry, and clinical institutions to provide a radically open access data platform accessible by researchers around the world.

### Approval for conducting the research

- MIMIC-IV study was reviewed by the Institutional Review Board at the Beth Israel Deaconess Medical Center, which granted a waiver of informed consent and approved the data-sharing initiative. The study adhered to all relevant institutional policies and ethical guidelines, maintaining strict confidentiality and security protocols for the MIMIC-IV data.

### Patient population

We used the MIMIC-IV database and included patients who were admitted to the ICU of any type at the Beth Israel Deaconess Medical Center between 2008 and 2019. The primary reason for their hospitalization was septicemia, which resulted in the development of sepsis. This was used to rule out all the patients that developed sepsis as a result of any medical procedures or other conditions which could have caused sepsis or septic shock and therefore influence the results. Patients whose clinical presentation of sepsis progressed towards septic shock were also included in the study. Diagnosis of sepsis and septic shock were established based on MIMIC-IV data that included all diagnoses billed to each patient at the end of their hospital stay coded using ICD-9 and ICD-10 codes. Since we aimed to identify septic patients as accurately as possible, we did not rely on any clinical parameters or other variables needed to establish whether a patient has sepsis/septic shock or not but instead used the pre-coded diagnoses made by clinicians in the electronic health records. Thus, we did not include in our study the details about which sepsis definition was used.

Since the MIMIC-IV study followed patient data over multiple years, some patients had more than one hospitalization in the intensive care units. To eliminate potential bias that could arise because of that, we included data for each patient only from their chronologically first hospitalization. As a result, 7546 patients were included with each patient having only one (chronologically first) hospitalization (Supplementary Fig. 1).

### Variables

#### General information

We have included the following demographic data for each patient: age at the time of the hospitalization, gender, and ethnicity. Furthermore, outcomes of the hospitalization (whether the patient was alive or dead at hospital discharge) and the length of the hospitalization were also obtained.

#### Laboratory analysis

For each patient, we obtained all records of laboratory measurements that were done during the hospitalization. Since some of the laboratory parameters were measured multiple times during a given patient’s hospital stay, we analyzed only the first chronologically available measurements to get the most accurate representation of the patient’s condition at ED or early hospitalization (EH). The data were then used to determine the overall hospital mortality risk. Based on this, the hospital staff would be able to triage septic patients early and promptly initiate treatment. First available measurements also correlate best with the patient’s condition before any medical intervention, and as such are optimal for the construction of a model that would predict mortality risk in patients with sepsis at the ED or in EH.

To ensure a statistically significant sample size, we selected only the laboratory findings which were measured at least once in at least 1000 patients. The final number of analyzed parameters was 75; the full list can be found in Supplementary Table 1.

All laboratory measurements were interpreted as normal, abnormally high, or abnormally low according to the reference range set by the MIMIC-IV study. In certain cases, reference ranges set by the MIMIC-IV study changed over the years for the same laboratory measurement, and hence some of the laboratory parameters have multiple reference ranges, all of which are listed in Supplementary Table 1 along with all reference ranges for all laboratory measurements that were used for the construction of e-SEPSS.

#### Comorbidities

To analyze to what extent the presence of comorbidities affects patients’ outcomes, we browsed the data that included all billed diagnoses to patients at the end of their hospitalization and extrapolated the comorbidity diagnoses for each included patient.

After obtaining all data, we observed many diagnoses and codes classified according to the International Classification of Disease versions 9 and 10 (ICD-9 and ICD-10). To gain a more comprehensive overview of the impact on patient outcomes, we grouped the comorbidities according to the ICD codes, instead of analyzing each comorbidity separately.

The final 13 groups were as follows:

- Liver diseases.
- Asthma.
- Chronic obstructive pulmonary disease (COPD).
- Renal failure.
- Neoplasms.
- Ischemic heart disease.
- Diseases of pulmonary circulation.
- Heart failure.
- Chronic rheumatic heart disease (CRHD).
- Hypertension.
- Cerebrovascular disorders.
- Diabetes.
- Hereditary and degenerative diseases of the central nervous system (CNS).

The table below contains all the ICD codes that were included in each comorbidity group (Table 1). To enhance language fluency, each of these comorbidity groups will be referred to as “comorbidity” instead of “comorbidity group” later in the article.

**Table 1:** List of all international classification of diseases (ICD) codes included in each comorbidity group

Comorbidity group	ICD-9 code	ICD-10 code
Liver diseases	571, 572, 573	K72.1, K72.9, K70, K71, K73, K74, K75, K76, K77
Asthma	493	J45
Chronic obstructive pulmonary disease	490, 491, 492, 494, 495, 496	J44
Renal failure	585, 586	N18, N19
Neoplasms	14, 15, 16, 17, 18, 19, 20, 21, 22, 23	C, D0, D1, D2, D3, D4
Ischemic heart disease	412, 413, 414	I25
Pulmonary circulation diseases	416, 417	I27, I28
Heart failure	428	I50
Chronic rheumatic heart disease	393, 394, 395, 396, 397,398	I0
Hypertension	401, 402, 403, 404, 405	I1
Cerebrovascular disorders	437, 438	I65, I66, I67, I68, I69
Diabetes	249, 250	E08, E09, E10, E11, E13
Hereditary and degenerative diseases of the central nervous system: multiple sclerosis, Alzheimer’s disease, amyotrophic lateral sclerosis, Parkinson’s disease	340, 331.0, 335.2, 332.0	G35, G30, G12.21, G20

#### Outcomes

For each patient, the MIMIC-IV study recorded whether the patient was alive or deceased at the end of the hospitalization and the duration of the hospitalization. We included no other outcomes for our study.

### Statistical analysis

For descriptive statistics, each numerical variable is presented using the median along with the interquartile range (IQR) due to significant deviation from the normal distribution. The significance of differences between covariates was determined by the Mann-Whitney U test for numerical, and the chi-squared test for categorical data.

Odds ratios for each of the 13 comorbidities and 75 laboratory parameters were calculated using univariable binary logistic regression to determine their association with in-hospital mortality. For the univariable analysis of laboratory parameters, both their increased and decreased levels were compared to normal levels and corresponding odds ratios were reported for increased and decreased levels separately. After the analysis of each of the covariates individually, we aimed to develop and validate the adjusted model for predicting the risk of in-hospital mortality using the combined effect of all laboratory parameters. For that purpose, we divided patients into two groups: model development (MD) and model validation (MV) group. All laboratory parameters that were statistically significant in univariable analysis (either their decreased or increased levels) were selected as covariates in the multiple binary logistic regression analysis which was used for the MD. Additionally, due to the high risk of bias, all laboratory parameters with no reported values for more than 50% of patients were excluded from the multiple regression analysis. Missing values in the variables included in the analysis were managed using listwise deletion of missing data. Therefore, the MD group consisted of all patients that had a reported value for all the laboratory parameters that were selected for the multiple regression analysis. Similar to the univariable analysis, for each laboratory parameter included in the MD, the effect of both their increased and decreased levels in comparison to normal levels was analyzed. The final set of mortality-predicting variables was determined with the forward stepwise logistic regression using the group of parameters selected for the MD. All variables with the *P*-values of less than 0.05 were considered statistically significant and were incorporated into the final prediction model and scoring system. All reported odds ratios were also adjusted for age and gender (adjusted odds ratio, aOR).

The final model was then used as a basis for the formation of the new scoring system for calculating the risk for in-hospital mortality for each patient. The points in the scoring system were obtained by rounding the regression coefficients of a variable to the nearest integer. For each patient, the total number of points was defined as the sum of points for all the abnormal laboratory parameters. In-hospital mortality was then assessed separately for each group of patients with a different number of points to establish whether the scoring system has sufficient power in differentiating patients with different mortality risks.

The validity of the model was evaluated using Receiver Operating Characteristic (ROC) curve and Hosmer-Lemeshow goodness-of-fit test. To assess the internal validity of the model, all patients who were not included in the MD group were used as a model validation group. Predicted mortality, i.e., mortality in the MD group was then compared with mortality in the validation group to determine if the scoring system can accurately predict mortality in a cohort of patients that were not a part of model construction. This internal validation analysis was done separately for each group of patients with a different number of points and the chi-squared test was used to assess whether mortality in the validation group was statistically different from the predicted mortality, thus testing the prediction accuracy of the model. Also, the Cochran-Armitage test was used to confirm the linear association between a higher number of points and higher in-hospital mortality which would corroborate previous evidence of the overall validity of the model. Finally, to establish potential differences in survival time between groups of patients with a different number of points, the Kaplan-Meier analysis was used. The significance level for all statistical tests was set to 0.05.

### Software and data source

All data for this study was obtained from the MIMIC-IV database, version 1.0. For searching and extracting data from the MIMIC-IV database, Microsoft SQL Server Management Studio 18 was used (Microsoft Corp., Redmond, Washington, USA). The acquired data was statistically analyzed using IBM SPSS Statistics, version 20 (IBM Corp., Armonk, New York, USA). GraphPad Prism version 8.4.2 (GraphPad Software, San Diego, California, USA) was used for all graphical visualization of the data.

## Results

**Fig. 1:**
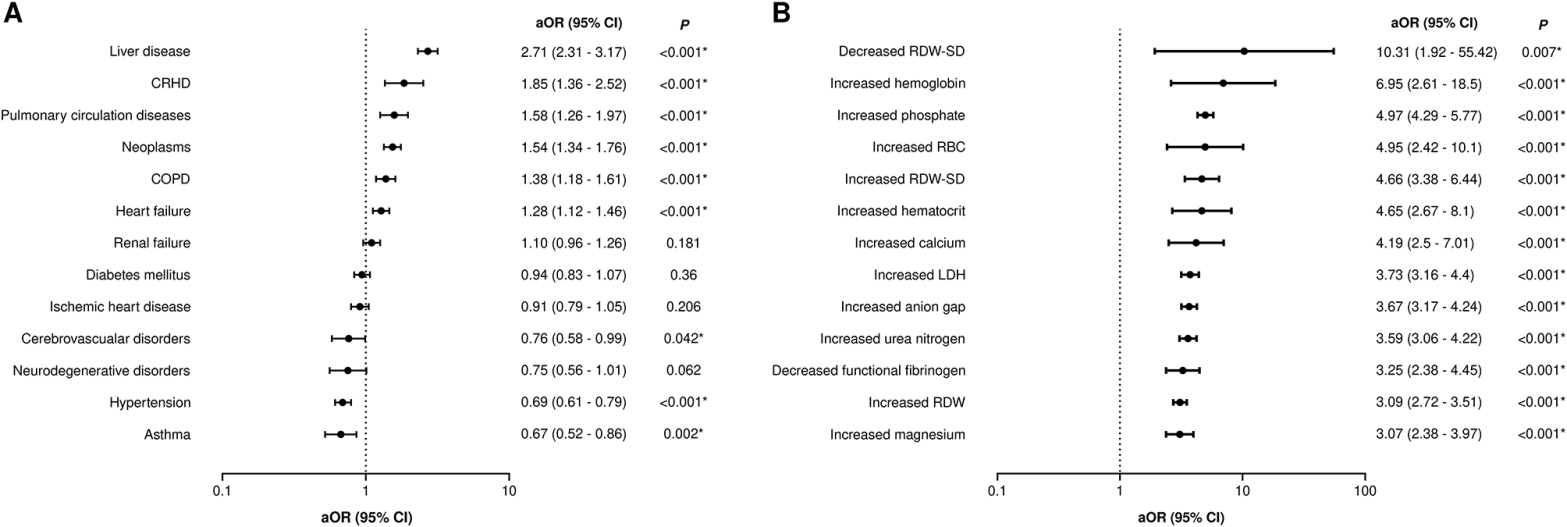
Adjusted odds ratios (aOR) for all comorbidities (**A**) and laboratory parameters (**B**) with the highest aORs. 95% CI − 95% confidence interval, CRHD - chronic rheumatic heart disease, COPD - chronic obstructive pulmonary disease, RDW-SD - red cell distribution width - standard deviation, RBC - red blood cells, LDH - lactate dehydrogenase, * - significant *P*-values

The study included 7546 patients with the diagnosis of septicemia which resulted in the development of sepsis. 51.4% of patients were male, 68.2% were white, and the median age was 70 (57–82). We then compared these demographic characteristics between the groups of patients who survived (83.4%) and those who died (16.6%) (Table 2). Our results show that patients who survived were significantly younger compared to those who died (69; 57–81 vs. 74; 61–85, p-value < 0.001) with no significant differences among genders. When we compared the median hospital duration, we showed that patients who survived were hospitalized 2 days longer (7; 4–11 vs. 5; 2–10, p-value < 0.001). The group of patients who died had both a higher proportion of patients with at least one comorbidity (95.2% vs. 88.5%, p-value < 0.001) and also a higher median number of comorbidities per one patient (3; 2–4 vs. 2; 1–4, p-value < 0.001). Univariable logistic regression showed that liver diseases and CRHD were the greatest mortality risk factors with aOR of 2.71 (95% C.I.: 2.31–3.17, p-value < 0.001) and 1.85 (95% C.I.: 1.36–2.52, p-value < 0.001), respectively (Fig. 1B).

**Table 2:** Patients’ demographic characteristics

		Overall survival			
		All	Yes	No	
		*N* (%)	*N* (%)	*N* (%)	*P*
		7546 (100)	6295 (83.4)	1251 (16.6)	
**Age**	18–65	2888 (38.3)	2503 (39.8)	385 (30.8)	< 0.001*
	≥ 65	4658 (61.7)	3792 (60.2)	866 (69.2)	
**Gender**	Male	3876 (51.4)	3225 (51.2)	651 (52)	0.602
	Female	3670 (48.6)	3070 (48.8)	600 (48)	
**Ethnicity**	White	5144 (68.2)	4327 (68.7)	817 (65.3)	< 0.001*
	Black / African American	951 (12.6)	831 (13.2)	120 (9.6)	
	Asian	301 (4)	256 (4.1)	45 (3.6)	
	Hispanic / Latino	303 (4)	270 (4.3)	33 (2.6)	
	Other or unable to obtain	847 (11.2)	611 (9.7)	236 (18.9)	
**Hospitalization duration**	≤ 10 days	5658 (75)	4709 (74.8)	949 (75.9)	0.606
	11–30 days	1714 (22.7)	1438 (22.8)	276 (22.1)	
	≥ 31 days	173 (2.3)	148 (2.4)	25 (2.0)	
**Comorbidities**	Any comorbidity present	6765 (89.7)	5574 (88.5)	1191 (95.2)	< 0.001*
	Hypertension	4698 (62.3)	3943 (62.6)	755 (60.4)	0.128
	Cerebrovascular disorders	469 (6.2)	400 (6.4)	69 (5.5)	0.262
	Diabetes mellitus	2542 (33.7)	2122 (33.7)	420 (33.6)	0.926
	Neoplasms	1833 (24.3)	1438 (22.8)	395 (31.6)	< 0.001*
	Ischemic heart disease	1759 (23.3)	1449 (23)	310 (24.8)	0.178
	Diseases of pulmonary circulation	468 (6.2)	353 (5.6)	115 (9.2)	< 0.001*
	Heart failure	2145 (28.4)	1696 (26.9)	449 (35.9)	< 0.001*
	Chronic rheumatic heart disease	210 (2.8)	149 (2.4)	61 (4.9)	< 0.001*
	Liver disease	1029 (13.6)	745 (11.8)	284 (22.7)	< 0.001*
	Asthma	666 (8.8)	590 (9.4)	76 (6.1)	< 0.001*
	Chronic obstructive pulmonary disease	1153 (15.3)	900 (14.3)	253 (20.2)	< 0.001*
	Renal failure	1928 (25.5)	1560 (24.8)	368 (29.4)	0.001*
	Hereditary and degenerative diseases of the central nervous system	364 (4.8)	309 (4.9)	55 (4.4)	0.440

* - significant *P*-values

In order to create our mortality-predicting model, we focused on values of all available laboratory parameters. The values of most of them (64/75 analyzed, 85.3%) were significantly different between patients who survived and those who died (Supplementary Table 2). Four parameters had no reported cut-off values and were therefore excluded from further analyses as we could not determine the normal, decreased and increased levels of that parameter. For each of the remaining 71 laboratory parameters, we analyzed the impact on mortality of both increased and decreased levels compared to normal levels. After performing a univariable logistic regression, our results demonstrated that 66 laboratory parameters (93.0%) were significant mortality risk factors (Supplementary Table 3) (Fig. 1A).

From those 66 parameters, we included 40 in further analyses because the remaining parameters were not reported in more than 50% of patients. For each of the 40 parameters, we assessed the impacts of both their increased and decreased values on overall mortality. The model (which would become the e-SEPSS scoring system) was then constructed on 1004 patients (model development group, MD) using multiple binary logistic regression (Table 3 and Supplementary Table 4). The final model yielded 7 parameters which were the most significant predictors of death: decreased chlorides, increased mean corpuscular hemoglobin (MCH), increased red blood cell distribution width (RDW), increased phosphates, increased partial thromboplastin time (PTT), increased lactate dehydrogenase (LDH), and decreased blood pH.

**Table 3:** Development and validation of the e-SEPSS model for predicting mortality

Predictor variable	Cut-off values	Regression coefficients	aOR (95% CI)	*P*	e-SEPSS points
Decreased chlorides	< 96 mEq/L	0.496	1.643 (1.109–2.432)	0.013*	1
Increased MCH	> 32 pg	0.665	1.945 (1.339–2.825)	< 0.001*	1
Increased RDW	> 15.5%	0.812	2.253 (1.610–3.154)	< 0.001*	1
Increased phosphates	> 4.5 mg/dL	0.640	1.896 (1.286–2.795)	0.001*	1
Increased PTT	> 35 or > 36.5 s**	0.793	2.209 (1.577–3.095)	< 0.001*	1
Increased LDH	> 250 IU/L	0.821	2.273 (1.597–3.236)	< 0.001*	1
Decreased pH	< 7.35 units	0.595	1.813 (1.272–2.584)	0.001*	1

The model was statistically significant (χ^2^(20) = 169.11, *P* < 0.001), with 22.7% of explained variance (Nagelkerke R [2]) and 76.7% of correctly classified cases

MCH - mean corpuscular hemoglobin, RDW - red blood cell distribution width, PTT - partial thromboplastin time, LDH - lactate dehydrogenase, * - significant *P*-values, ** - PTT has two different reference ranges as described in the Methods section

**Fig. 2:**
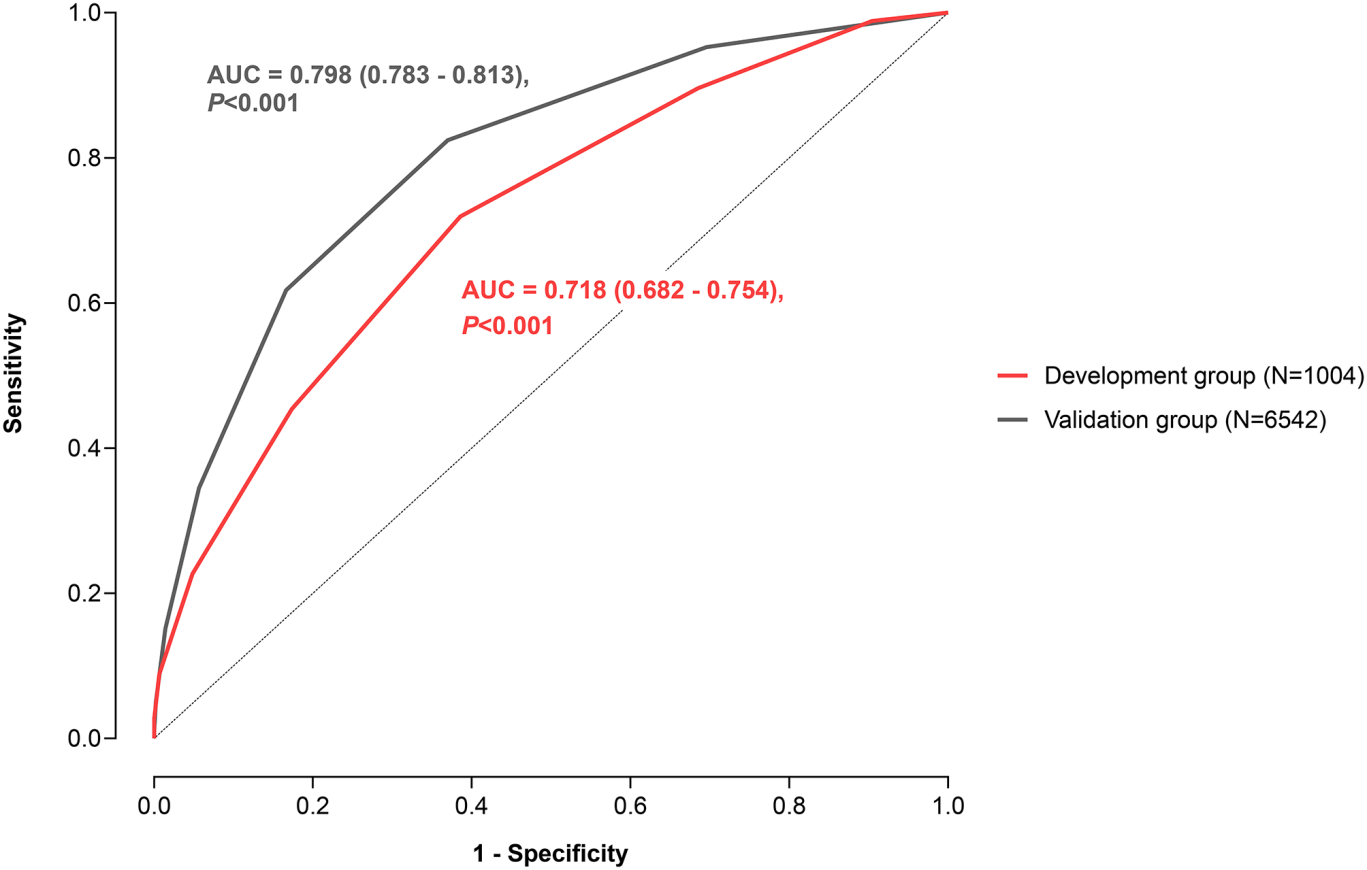
ROC curve analysis for the model development and model validation groups

The 6542 patients that were not included in the MD were used as a model validation group (MV). Results of the ROC curve analysis showed overall satisfactory discriminative power in both the development and validation groups (Fig. 2). The model’s validity was further evaluated with the Hosmer-Lemeshow test which showed satisfactory goodness of fit (p-value > 0.05) and demonstrated that observed mortality matched the one predicted by our model using these 7 parameters.

**Fig. 3:**
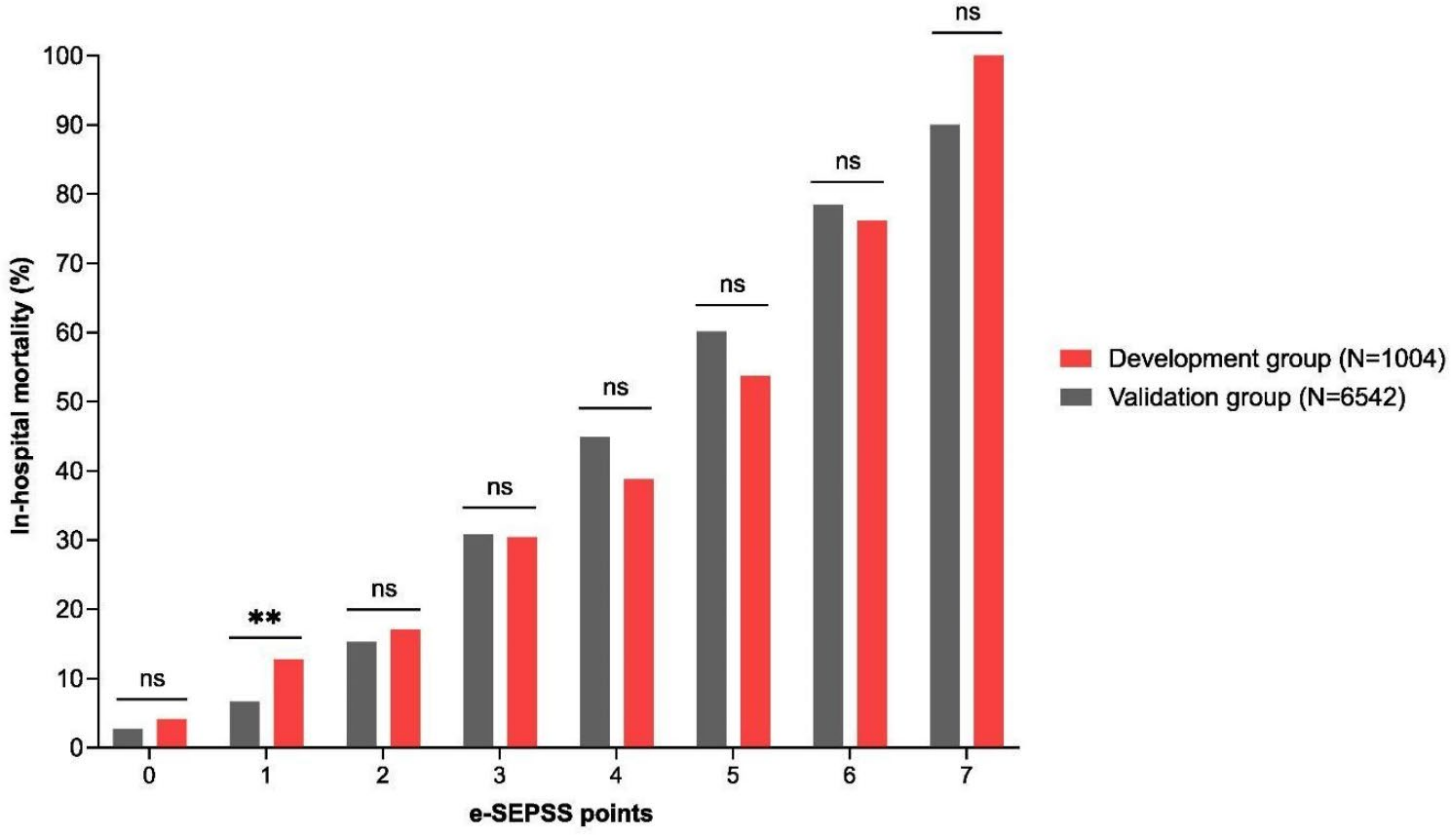
In-hospital mortality stratified according to the number of e-SEPSS points. ns - not significant, ** - *P* = 0.001

Those 7 parameters were then used to create a new scoring system to predict overall mortality in patients with sepsis at ED and during EH. The final scoring system e-SEPSS has a maximum of seven points with one point given to each of the seven risk factors. Then, comparison of the overall mortality between groups of patients with different numbers of points was performed on a sample of 1004 patients from the MD group. Results show a significant and robust linear trend of increasing risk of mortality with an increasing number of points (Cochran-Armitage chi-square, p-value < 0.001), which suggests that e-SEPSS has significant ability in differentiating patients by their risk of mortality (Fig. 3, Supplementary Table 5). The group of patients with zero points had the lowest mortality of only 4.1% and from that level, the mortality consistently grew higher with each additional point and reached a mortality of 53.7% in the group of patients with 5 points. Then, the mortality continued to increase at even higher rates with the group of patients with 6 points where the mortality was 76.2%. Finally, the maximum mortality of 100% was reached in the group of patients with all 7 points, i.e., with all 7 risk factors present, in which all patients died. Similar to the MD group, the results in the MV group (which served as an internal validation group) show a consistent linear increase in mortality with each additional point (Cochran-Armitage chi-square, p-value < 0.001). In addition, the differences in mortality between the MD and MV groups were not statistically significant for any of the point groups except for the group of patients that had 1 point (p-value = 0.001). Therefore, our internal validation suggested that in this group of 6542 patients that were not part of MD, e-SEPSS also predicted mortality precisely and with no significant deviations from predictions established in the MD group. This further supports the overall high validity of our model. Overall differences between MD and MV groups are represented in Supplementary Table 6.

Finally, we wanted to establish if there were any differences in the duration of hospitalization between patients with a different number of points. The shortest time spent in the hospital was observed in patients with 0 and 7 points with an average of 6.3 and 4.2 days, respectively (Supplementary Fig. 2). Surprisingly, the longest overall duration of hospitalization was observed among patients with 5 points (10.7 days) and was roughly similar in patients with 3 and 4 points. Also, Kaplan-Meier curve analysis was performed in the group of patients who died to establish the differences in their survival time. There was a significant trend of decreased time-to-death for patients with a higher number of points (Mantel-Cox χ^2^(7) = 994.2, p-value < 0.001), with an estimated survival time nearly 19 times longer in patients with zero points compared to patients with the maximum seven points (Supplementary Fig. 3).

Taken together, these results suggest the high reliability of e-SEPSS in predicting overall in-hospital mortality from data taken at ED or EH and high discriminative power in differentiating patients with different mortality risks.

## Discussion

Sepsis is a severe medical condition with a substantial global health burden [6]. Prompt diagnosis and timely treatment are crucial to reduce mortality rates [27]. However, the complex and multifactorial nature of sepsis makes it challenging for physicians to reliably triage patients into severity-based groups at hospital admission [28–30]. In this study, we developed and validated a novel scoring system, e-SEPSS, designed to predict in-hospital mortality risk for patients with sepsis at the emergency department and early hospital admission. The scoring system is based on 7 commonly used blood parameters, allowing for a rapid and reliable screening method that can be used at admission to efficiently triage patients based on the predicted risk of sepsis mortality and initiate prompt and adequate treatment. Our results from the model development group demonstrate that e-SEPSS effectively stratifies patients into 8 mortality risk groups based on the number of points they receive (0–7 points), with a linear increase in mortality from the minimal risk group to the group with highest risk of death (and the maximum e-SEPSS points).

We conducted an internal validation with 6542 patients that were not included in the e-SEPSS development group to assess the reliability of our scoring system in a different group of patients. Our analysis showed the same linear trend, with mortality ranging from 2.7% (0 points) to 90% (7 points). Even though only the group with 1 point was statistically different in terms of mortality between the development and validation groups, it did not skew the linear increase in mortality as the number of points increased, thus showing the reliability of the model. However, an external validation in independent cohorts is warranted to further validate the performance of e-SEPSS and enhance its clinical utility in real-world settings.

Several scoring systems are used to determine the general severity-of-illness score of adults such as APACHE, SOFA, and SAPS [31, 32]. However, all of them have limitations in predicting sepsis mortality in the very early stages such as the emergency department admission. The mean time for introducing a patient’s data to APACHE IV is longer than half an hour and because of the necessity of gathering data over 24 h, the system is troublesome and prone to mistakes [33]. Calculating SOFA is also time-consuming since it requires several clinical and laboratory parameters over 24 h that reflect the dysfunction of several organs [34]. Finally, SAPS is designed from the beginning as a simpler score, but primarily after admission to the ICU, similar to the and SOFA [35–37]. On the other hand, laboratory values required to calculate e-SEPSS can be obtained within a few hours, potentially allowing for faster assessment and decision-making.

Existing scoring systems could also be influenced by subjective clinical bias. For example, Polderman et al. showed that there was a 15% variability in the score when the same physicians’ calculated APACHE 4 months apart, arguing that it has inherent score variability [38]. Moreover, a review showed that several factors may influence the reliability of the Glasgow Coma Scale score which is a part of APACHE, SOFA, and SAPS, including education and training, the level of consciousness, and the type of stimuli used, thus suggesting that different medical personnel may obtain different scores which might impact the overall result [39]. By relying solely on objective laboratory values, e-SEPSS aims to reduce the inter-observer variability.

While APACHE, SAPS, or SOFA are reliable for assessing general severity-of-illness scores in adult intensive care units, they are not specific for patients with sepsis [40]. We developed and internally validated e-SEPSS specifically for sepsis patients, addressing the reduced sepsis-specificity in general severity-of-illness scores, but it has yet to be tested in patients with other illnesses. In a real-world scenario, we believe that calculating e-SEPSS should be quick since all 7 blood parameters are considered a part of a standard clinical workup that can be performed relatively quickly. Adding to this, e-SEPSS provides accessibility and affordability for almost any healthcare facility worldwide due to requiring only 7 blood parameters. By relying solely on numerical laboratory values, e-SPESS eliminated the need for subjective clinical evaluations and optimizes objectiveness when scoring. Because of these reasons, we believe e-SEPSS complements, rather than replaces any of the already used scoring systems since they have a considerable clinical benefit after emergency department admission and the earliest hospitalization period, especially in the ICU. Therefore, the addition of e-SEPSS to clinical practice after external validations has the potential to bridge this important time gap between early admission and the ICU.

Despite the somewhat different purpose of APACHE, SOFA, or SAPS and e-SEPSS (general severity of illness vs. predicting mortality risk during early stages of sepsis), we wanted to compare the predictive value of the aforementioned scoring systems in terms of assessing mortality risk. We performed a literature search of recent publications that assessed the value in predicting mortality in patients with sepsis and also reported their value of area under the ROC curve (AUC) for the 4 scoring systems. AUC is commonly used in assessing the accuracy and discrimination performance of scoring systems [41]. We note that we did not use a direct performance analysis (i.e. directly comparing e-SEPSS to the aforementioned scoring systems) since scores for APACHE, SOFA and SAPS could not be obtained from the MIMIC-IV database and the different way they are scored makes it difficult to compare the results directly to e-SEPSS. Nevertheless, we used AUC to provide a comparative assessment of the predictive performance of e-SEPSS against these established scoring systems in the context of sepsis mortality prediction.

Results of a large review from 2008 showed that the overall value of SOFA score in predicting mortality in sepsis patients specifically on the day of hospital admission measured by AUC was between 0.61 and 0.88 [42]. Our model also had a high predictive value with an AUC of 0.798 (Fig. 2), which may imply non-inferiority to the SOFA score in determining mortality risk, but without a direct performance analysis it is difficult to make definitive comparisons. More recent studies also showed similar or even lower AUCs for SOFA scores when compared to e-SEPSS, suggesting that e-SEPSS had promising predictive capability [39, 43–60]. Although comprehensive systematic reviews are still missing, the APACHE II score has AUC values in the range of 0.64 to 0.86 based on several recent studies [45, 47, 49, 51, 52, 54–56, 59, 61–63]. One study also reported an AUC of 0.743 for the APACHE II score specifically for mortality at ICU admission [55]. SAPS II had a similar range of 0.60 to 0.85, although systematic reviews and meta-analyses of its AUC are also not available [48, 49, 51, 52, 63–65]. Taken together, these results suggest that, when compared to other prediction models in terms of AUC, e-SEPSS has promising predictive capability, warranting further validation through direct comparative studies.

The study has several limitations that should be acknowledged. Firstly, it was conducted in a single center, which may limit the generalizability of the findings to other healthcare settings. Additionally, the retrospective design introduces potential biases and confounding factors, highlighting the need for prospective studies. Secondly, regarding laboratory measurements analysis, we have encountered a lot of missing values, i.e., a patient would have a specific laboratory parameter measurement requested, but the results were not available in the database, thus reducing the pool of available results for the construction of our model. Additionally, we recognize that in some resource-limited medical settings, obtaining laboratory results may take more than a few hours, which could delay the e-SEPSS calculation. The small sample and large proportion of missing data size in our study may affect the statistical power and precision of the results, emphasizing the importance of larger sample sizes for more reliable conclusions. Missing data was managed using listwise deletion which could also lead to loss of statistical power, but in the setting of a large proportion of missing values, we found the method most suitable with the most reduced amount of bias. Furthermore, the exclusion of other relevant clinical variables, such as comorbidities or microbiological data, may limit the comprehensive predictive capability of the e-SEPSS scoring system. Despite promising results from our internal validation, an external validation using independent cohorts from different healthcare settings is necessary to confirm the predictive accuracy and applicability of e-SEPSS. Since we did not stratify patients by multiple definitions of sepsis (Sepsis-1, Spesis-2 and Sepsis-3) [66], our scoring system might be less or more predictable in certain patients, thus a more comprehensive investigation is highly encouraged. Also, our study focused on mortality as the main measure of outcome because it provides a broad understanding of intervention impact and disease severity, but future research could adopt a multidimensional approach, incorporating both short-term clinical outcomes and in-hospital mortality, to provide a comprehensive evaluation of interventions and guide clinical practices. While our model identifies several factors that significantly predict sepsis mortality, the causal relationship between these factors and outcomes remains uncertain. As Zhang et al. suggest, causal inference in observational data is challenging due to the potential for confounding and bias [67]. Marginal structural models (MSMs) and inverse probability weighting (IPW) offer robust approaches for addressing these challenges by accounting for time-varying confounders. Applying these methods in future studies could help elucidate the true causal pathways underlying sepsis outcomes, improving clinical decision-making and patient management. While e-SEPSS has been designed to be quick to calculate from only 7 blood parameters, we acknowledge that in specific environments blood results may take somewhat longer to obtain. Lastly, ethical considerations surrounding the implementation of the scoring system in clinical practice, such as resource allocation and patient care decisions, need to be carefully evaluated.

To summarize, our developed scoring system, e-SEPSS, offers a reliable, quick, simple, and cost-effective approach to stratify patients with sepsis based on their mortality risk at the emergency department or very early hospital admission (e.g. in a medical ward), before the patients enter the ICU. This method could significantly shorten the period from patient evaluation to initiation of treatment, which has been proven key for sepsis outcomes. Our scoring system is based on 7 commonly tested blood laboratory parameters and has been validated in over 7500 patients, showing a linear increase in mortality from the group with zero points (minimal mortality risk) to the group with the maximum 7 points. Furthermore, it allows the staff of almost any hospital with minimal equipment to assess the mortality risk of a patient at ED and EH and appropriately allocate hospital resources. Finally, after future external clinical validation, our scoring system might be a good addition to the already existing scoring systems such as APACHE, SOFA, and SAPS, all while offering clinicians another useful tool in reducing the significant health and economic burden of sepsis.

## Electronic supplementary material

Below is the link to the electronic supplementary material.

Supplementary Material 1

## Data Availability

Data used in this study can be found in MIMIC-IV database.

## Abbreviations

95% CI: 95% confidence interval
aOR: Adjusted odds ratio
APACHE: Acute Physiology and Chronic Health Evaluation
AUC: Area under the ROC curve
CNS: Central nervous system
COPD: Chronic obstructive pulmonary disease
CRHD: Chronic rheumatic heart disease
ED: Emergency department
EH: Early hospitalization
e-SEPSS: Early SEpsis mortality Prediction Scoring System
ICD: International Classification of Disease
ICU: Intensive care unit
INR/PT: International normalized Ratio / prothrombin time
IQR: Interquartile range
MCH: Mean corpuscular hemoglobin
MCHC: Mean corpuscular haemoglobin concentration
MCV: Mean corpuscular volume
MD: Model development
MIMIC: Medical Information Mart for Intensive Care
MV: Model validation
NT-proBNP: N-terminal proBrain Natriuretic Peptide
PT: Prothrombin time
PTT: Partial thromboplastin time
RDW: Red cell distribution width
RDW-SD: Red cell distribution width-standard deviation
ROC: Receiver operating characteristic
SAPS: Simplified Acute Physiology Score
SOFA: Sequential Organ Failure Assessment
