# Supplementary material for: A novel scoring system for evaluating mortality risk of patients with sepsis during early hospitalization
Source: BMC Infect Dis. 2025 Jul 1;25:876. doi: 10.1186/s12879-025-10920-8 (PMC12219925; doi:10.1186/s12879-025-10920-8)
Supplement: Supplementary file 1 — Supplementary Material 1 [file 12879_2025_10920_MOESM1_ESM.docx]

**Supplementary Table 1.** Cut-off values for all laboratory parameters extracted from the MIMIC-IV study and included in the final analysis

| **Laboratory parameter** | **Sample** | **Units** | **Upper cut-off value** | **Lower cut-off value** |
| --- | --- | --- | --- | --- |
| Absolute Basophil Count | Blood | K/uL | 0.01 | 0.08 |
| Absolute Eosinophil Count | Blood | K/uL | 0.04 | 0.54 |
| Absolute Lymphocyte Count | Blood | K/uL | 1.2 | 3.7 |
| Absolute Monocyte Count | Blood | K/uL | 0.2 | 0.8 |
| Absolute Neutrophil Count | Blood | K/uL | 1.6 | 6.1 |
| Alanine Aminotransferase | Blood | IU/L | 0 | 40 |
| Albumin | Blood | g/dL | 3.4, 3.5 | 4.8, 5.2 |
| Alkaline Phosphatase | Blood | IU/L | 35, 39, 40 | 105, 117, 130 |
| Anion Gap | Blood | mEq/L | 8, 10 | 16, 18, 20 |
| Aspartate Aminotransferase | Blood | IU/L | 0 | 40 |
| Atypical Lymphocytes | Ascites | % | 0 | 0 |
| Bands | Ascites | % | 0 | 5 |
| Base Excess | Blood | mEq/L | *no reported cut-off values* | |
| Basophils | Ascites | % | 0 | 1, 2 |
| Bicarbonate | Blood | mEq/L | 22 | 32 |
| Bilirubin, Direct | Blood | mg/dL | 0 | 0.3 |
| Bilirubin, Indirect | Blood | mg/dL | *no reported cut-off values* | |
| Bilirubin, Total | Blood | mg/dL | 0 | 1.5 |
| Calcium, Total | Blood | mg/dL | 8.4 | 10.2, 10.3 |
| Calculated Total CO_2_ | Blood | mEq/L | 21 | 30 |
| Chloride | Blood | mEq/L | 96 | 108 |
| Cortisol | Blood | ug/dL | 2 | 20 |
| C-Reactive Protein | Blood | mg/L | 0 | 5 |
| Creatine Kinase | Blood | IU/L | 26, 29, 38, 47 | 140, 174, 201, 322 |
| Creatine Kinase, MB Isoenzyme | Blood | ng/mL | 0 | 10 |
| Creatinine | Blood | mg/dL | 0.4, 0.5 | 1.1, 1.2 |
| Eosinophils | Ascites | % | 0, 1 | 4, 7 |
| Ferritin | Blood | ng/mL | 13, 30 | 150, 400 |
| Fibrinogen, Functional | Blood | mg/dL | 150, 180 | 400 |
| Free Calcium | Blood | mmol/L | 1.12 | 1.32 |
| Glucose, blood gas | Blood | mg/dL | 70 | 105 |
| Glucose | Blood | mg/dL | 70 | 100, 105 |
| Haptoglobin | Blood | mg/dL | 30 | 200 |
| Hematocrit | Blood | % | 34, 36, 40 | 45, 48, 51, 52 |
| Hemoglobin | Blood | g/dL | 11.2, 12, 13.7, 14 | 15.7, 16, 17.5, 18 |
| Immature Granulocytes | Blood | % | 0 | 0.6 |
| International Normalized Ratio / Prothrombin Time (INR/PT) | Blood | / | 0.9 | 1.1 |
| Iron | Blood | ug/dL | 30, 45 | 160, 160 |
| Iron Binding Capacity, Total | Blood | ug/dL | 260 | 470 |
| Lactate | Blood | mmol/L | 0.5 | 2 |
| Lactate Dehydrogenase (LDH) | Blood | IU/L | 94 | 250 |
| Lipase | Blood | IU/L | 0 | 60 |
| Lymphocytes | Ascites | % | 18, 19 | 42, 53 |
| Magnesium | Blood | mg/dL | 1.5, 1.6 | 2.2, 2.6 |
| Mean Cellular Haemoglobin (MCH) | Blood | pg | 26, 27 | 32 |
| Mean Cellular Haemoglobin Concentration (MCHC) | Blood | g/dL | 31, 32 | 35, 37 |
| Mean Corpuscular Volume (MCV) | Blood | fL | 82 | 98 |
| Metamyelocytes | Ascites | % | 0 | 0 |
| Monocytes | Ascites | % | 2, 5 | 11, 13 |
| Myelocytes | Ascites | % | 0 | 0 |
| Neutrophils | Blood | % | 34, 50 | 70, 71 |
| N-terminal proBrain Natriuretic Peptide (NT-proBNP) | Blood | pg/mL | 0 | 93, 138, 177, 178, 192,  226, 229, 353, 624, 852 |
| Oxygen | Blood | % | *no reported cut-off values* | |
| Oxygen Saturation | Blood | % | *no reported cut-off values* | |
| pCO_2_ | Blood | mmHg | 35 | 45 |
| pH | Blood | units | 7.35 | 7.45 |
| Phosphate | Blood | mg/dL | 2.7 | 4.5 |
| Platelet Count | Blood | K/uL | 150 | 400, 440 |
| pO_2_ | Blood | mmHg | 85 | 105 |
| Potassium | Other | mEq/L | 3.3, 3.5 | 5.1, 5.4 |
| Potassium, Whole Blood | Blood | mEq/L | 3.3, 3.5, 3.5 | 5.1, 5.3, 5.4 |
| Prothrombin Time (PT) | Blood | sec | 9.4, 10.4 | 12.5, 13.4 |
| Partial Thromboplastin Time (PTT) | Blood | sec | 22, 25 | 35, 36.5 |
| Red Cell Distribution Width (RDW) | Blood | % | 10.5 | 15.5 |
| Red Cell Distribution Width - Standard Deviation (RDW-SD) | Blood | fL | 35.1 | 46.3 |
| Red Blood Cells | Blood | m/uL | 3.9, 4.2, 4.6 | 5.2, 5.4, 6.1, 6.2 |
| Reticulocyte Count, Automated | Blood | % | 0.4, 1.2 | 2, 3.2 |
| Sodium | Blood | mEq/L | 133, 135 | 145, 147 |
| Sodium, Whole Blood | Blood | mEq/L | 133, 135, 135 | 145, 147, 148 |
| Thyroid Stimulating Hormone | Blood | uIU/mL | 0.27 | 4.2 |
| Transferrin | Blood | mg/dL | 200 | 360 |
| Troponin T | Blood | ng/mL | 0 | 0.01 |
| Urea Nitrogen | Blood | mg/dL | 6 | 20 |
| Vitamin B12 | Blood | pg/mL | 240 | 900 |
| White Blood Cells | Blood | K/uL | 4 | 10, 11 |

**Supplementary Table 2.** Differences in laboratory findings between patients who survived and died

| **Laboratory parameters** | **All** | | **Hospitalization outcome** | | | | |
| --- | --- | --- | --- | --- | --- | --- | --- |
|  |  |  | **Alive** | | **Dead** | | ***P-value*** |
|  | **N** | **MEDIAN (IQR)** | **N** | **MEDIAN (IQR)** | **N** | **MEDIAN (IQR)** |  |
| Absolute Basophil Count | 2644 | 0.02 (0 - 0.04) | 2259 | 0.02 (0 - 0.04) | 385 | 0 (0 - 0.03) | <0.001* |
| Absolute Eosinophil Count | 2645 | 0.01 (0 - 0.11) | 2260 | 0.01 (0 - 0.11) | 385 | 0.01 (0 - 0.1) | 0.011* |
| Absolute Lymphocyte Count | 2644 | 0.885 (0.49 - 1.42) | 2259 | 0.89 (0.5 - 1.43) | 385 | 0.83 (0.39 - 1.38) | 0.03* |
| Absolute Monocyte Count | 2644 | 0.75 (0.41 - 1.13) | 2259 | 0.75 (0.41 - 1.12) | 385 | 0.71 (0.38 - 1.25) | 0.938 |
| Absolute Neutrophil Count | 2644 | 10.205 (5.92 - 15.45) | 2259 | 9.88 (5.75 - 14.995) | 385 | 12.11 (7.22 - 18.36) | <0.001* |
| Alanine Aminotransferase | 5769 | 29 (16 - 64) | 4703 | 28 (16 - 60) | 1066 | 35 (18 - 91) | <0.001* |
| Albumin | 4555 | 2.9 (2.5 - 3.3) | 3693 | 3 (2.6 - 3.4) | 862 | 2.6 (2.2 - 3) | <0.001* |
| Alkaline Phosphatase | 5783 | 97 (69 - 154) | 4711 | 94 (68 - 148) | 1072 | 108 (75 - 184) | <0.001* |
| Anion Gap | 7513 | 15 (13 - 18) | 6284 | 15 (13 - 17) | 1229 | 17 (14 - 21) | <0.001* |
| Aspartate Aminotransferase | 5812 | 39 (23 - 84) | 4735 | 36 (22 - 72) | 1077 | 58 (30 - 169) | <0.001* |
| Atypical Lymphocytes | 2448 | 0 (0 - 0) | 1828 | 0 (0 - 0) | 620 | 0 (0 - 0) | 0.27 |
| Bands | 2619 | 2 (0 - 7) | 1957 | 2 (0 - 7) | 662 | 3 (0 - 8) | <0.001* |
| Base Excess | 4154 | -2 (-6 - 0) | 3010 | -1 (-5 - 0) | 1144 | -5 (-10 - 0) | <0.001* |
| Basophils | 5336 | 0.1 (0 - 0.3) | 4375 | 0.2 (0 - 0.3) | 961 | 0 (0 - 0.2) | <0.001* |
| Bicarbonate | 7510 | 22 (19 - 25) | 6281 | 22 (20 - 25) | 1229 | 20 (16 - 24) | <0.001* |
| Bilirubin, Direct | 952 | 1.9 (0.7 - 4) | 699 | 1.5 (0.55 - 3.45) | 253 | 2.9 (1.3 - 5.8) | <0.001* |
| Bilirubin, Indirect | 949 | 0.8 (0.4 - 1.6) | 706 | 0.7 (0.4 - 1.375) | 243 | 1.2 (0.6 - 3.55) | <0.001* |
| Bilirubin, Total | 5793 | 0.7 (0.4 - 1.5) | 4721 | 0.6 (0.4 - 1.3) | 1072 | 1 (0.4 - 2.9) | <0.001* |
| Calcium, Total | 7393 | 8.2 (7.6 - 8.7) | 6168 | 8.2 (7.7 - 8.7) | 1225 | 7.9 (7.3 - 8.6) | <0.001* |
| Calculated Total CO_2_ | 4154 | 23 (19 - 27) | 3010 | 24 (20 - 28) | 1144 | 21 (17 - 26) | <0.001* |
| Chloride | 7509 | 103 (99 - 107) | 6279 | 103 (99 - 107) | 1230 | 103 (97 - 108) | 0.121 |
| Cortisol | 849 | 22 (14.1 - 35.3) | 583 | 20.2 (12.85 - 29.75) | 266 | 27.6 (17.425 - 46) | <0.001* |
| C-Reactive Protein | 705 | 110.3 (52.8 - 182.5) | 627 | 106.3 (51.8 - 180.3) | 78 | 136.95 (68.55 - 186.875) | 0.112 |
| Creatine Kinase | 2641 | 101 (43 - 327) | 1969 | 98 (42 - 283) | 672 | 115 (47 - 424.25) | 0.008* |
| Creatine Kinase, MB Isoenzyme | 2395 | 4 (2 - 9) | 1738 | 4 (2 - 7) | 657 | 6 (3 - 13) | <0.001* |
| Creatinine | 7514 | 1.1 (0.8 - 1.9) | 6284 | 1.1 (0.8 - 1.7) | 1230 | 1.7 (1 - 2.8) | <0.001* |
| Eosinophils | 5336 | 0.2 (0 - 1) | 4375 | 0.2 (0 - 1.1) | 961 | 0.1 (0 - 1) | <0.001* |
| Ferritin | 1562 | 451 (202 - 956.75) | 1349 | 406 (190 - 852) | 213 | 844 (379 - 1755) | <0.001* |
| Fibrinogen, Functional | 1818 | 421 (253.25 - 588) | 1187 | 463 (320 - 625) | 631 | 302 (165.5 - 497.5) | <0.001* |
| Free Calcium | 2472 | 1.08 (1.02 - 1.143) | 1592 | 1.09 (1.03 - 1.15) | 880 | 1.06 (0.98 - 1.14) | <0.001* |
| Glucose | 1102 | 134 (104 - 187) | 669 | 139 (108 - 194) | 433 | 130 (96 - 176) | <0.001* |
| Glucose | 7508 | 121 (98 - 161) | 6279 | 120 (98 - 159) | 1229 | 124 (94 - 169) | 0.983 |
| Haptoglobin | 1474 | 217 (126 - 304) | 1155 | 225 (137 - 313.5) | 319 | 176 (85 - 264.5) | <0.001* |
| Hematocrit | 7481 | 32.2 (28 - 36.4) | 6258 | 32.3 (28.2 - 36.4) | 1223 | 31.4 (26.8 - 36.2) | <0.001* |
| Hemoglobin | 7490 | 10.5 (9 - 11.9) | 6268 | 10.5 (9.1 - 11.9) | 1222 | 10 (8.6 - 11.5) | <0.001* |
| Immature Granulocytes | 1952 | 0.7 (0.5 - 1.2) | 1738 | 0.7 (0.5 - 1.1) | 214 | 0.95 (0.6 - 1.6) | <0.001* |
| International Normalized Ratio / Prothrombin Time (INR / PT) | 6315 | 1.4 (1.2 - 1.8) | 5165 | 1.3 (1.2 - 1.7) | 1150 | 1.6 (1.3 - 2.3) | <0.001* |
| Iron | 1522 | 30 (17 - 53) | 1303 | 28 (16.5 - 49) | 219 | 39 (22 - 80) | <0.001* |
| Iron Binding Capacity, Total | 1478 | 183 (146 - 228) | 1270 | 190 (152 - 233) | 208 | 149.5 (108 - 191.25) | <0.001* |
| Lactate | 5548 | 1.9 (1.3 - 2.9) | 4359 | 1.8 (1.3 - 2.6) | 1189 | 2.6 (1.6 - 4.6) | <0.001* |
| Lactate Dehydrogenase (LDH) | 4484 | 251 (191 - 356.25) | 3579 | 235 (183 - 318) | 905 | 349 (250 - 644) | <0.001* |
| Lipase | 1812 | 27 (15 - 59) | 1445 | 27 (15 - 56) | 367 | 28 (13 - 73) | 0.934 |
| Lymphocytes | 5336 | 8 (4.175 - 13.7) | 4375 | 8.1 (4.45 - 14) | 961 | 7 (3.4 - 12) | <0.001* |
| Magnesium | 7420 | 1.9 (1.6 - 2.1) | 6196 | 1.8 (1.6 - 2.1) | 1224 | 2 (1.7 - 2.3) | <0.001* |
| Mean Cellular Haemoglobin (MCH) | 7489 | 29.9 (28.2 - 31.5) | 6267 | 29.9 (28.2 - 31.4) | 1222 | 30.15 (28.425 - 32.1) | <0.001* |
| Mean Cellular Haemoglobin Concentration (MCHC) | 7489 | 32.5 (31.4 - 33.5) | 6267 | 32.6 (31.5 - 33.6) | 1222 | 31.9 (30.7 - 33.1) | <0.001* |
| Mean Corpuscular Volume (MCV) | 7490 | 92 (87 - 96) | 6267 | 91 (87 - 96) | 1223 | 94 (89 - 100) | <0.001* |
| Metamyelocytes | 2502 | 0 (0 - 1) | 1862 | 0 (0 - 1) | 640 | 1 (0 - 2) | <0.001* |
| Monocytes | 5336 | 5 (3 - 7.8) | 4375 | 5.2 (3 - 8) | 961 | 4 (2.5 - 7) | <0.001* |
| Myelocytes | 2476 | 0 (0 - 0) | 1848 | 0 (0 - 0) | 628 | 0 (0 - 1) | <0.001* |
| Neutrophils | 5336 | 82.15 (73 - 88.3) | 4375 | 82.1 (73.5 - 88) | 961 | 82.6 (72 - 89.1) | 0.537 |
| N-terminal proBrain Natriuretic Peptide (NT-proBNP) | 864 | 4178.5 (1166.5 - 11449) | 686 | 3542 (1038.25 - 10064) | 178 | 6923.5 (2034.5 - 18200) | <0.001* |
| Oxygen | 1596 | 50 (40 - 98) | 916 | 50 (40 - 90) | 680 | 60 (50 - 100) | <0.001* |
| Oxygen Saturation | 2248 | 82 (68 - 95) | 1406 | 81 (68 - 95) | 842 | 84 (68 - 95) | 0.253 |
| pCO_2_ | 4154 | 40 (33 - 48) | 3010 | 39 (34 - 47) | 1144 | 41 (33 - 50) | 0.005* |
| pH | 4356 | 7.36 (7.28 - 7.41) | 3196 | 7.37 (7.31 - 7.42) | 1160 | 7.3 (7.21 - 7.38) | <0.001* |
| Phosphate | 7389 | 3.2 (2.6 - 4.1) | 6166 | 3.1 (2.5 - 3.8) | 1223 | 4.1 (3.1 - 5.7) | <0.001* |
| Platelet Count | 7477 | 188 (127 - 263) | 6257 | 191 (134 - 263) | 1220 | 170 (96.75 - 261.25) | <0.001* |
| pO_2_ | 4157 | 65 (43 - 102) | 3012 | 64 (42 - 102) | 1145 | 67 (44 - 103) | 0.097 |
| Potassium | 7508 | 4 (3.6 - 4.5) | 6277 | 4 (3.6 - 4.4) | 1231 | 4.3 (3.7 - 4.9) | <0.001* |
| Potassium, Whole Blood | 1576 | 4.1 (3.6 - 4.9) | 1015 | 4.1 (3.55 - 4.8) | 561 | 4.2 (3.6 - 5) | 0.005* |
| Prothrombin Time (PT) | 6319 | 15 (13.1 - 19.1) | 5167 | 14.7 (13 - 18) | 1152 | 17.45 (14.3 - 25.2) | <0.001* |
| Partial Thromboplastin Time (PTT) | 6178 | 32.2 (28.4 - 38.7) | 5034 | 31.6 (28.1 - 37) | 1144 | 36.65 (30.5 - 47.25) | <0.001* |
| Red Cell Distribution Width (RDW) | 7488 | 14.9 (13.7 - 16.7) | 6264 | 14.7 (13.6 - 16.3) | 1224 | 16.4 (14.8 - 18.3) | <0.001* |
| Red Cell Distribution Width - Standard Deviation (RDW-SD) | 3817 | 49.6 (45.2 - 55.8) | 3315 | 49 (44.9 - 54.5) | 502 | 55.8 (50.425 - 64.075) | <0.001* |
| Red Blood Cells | 7491 | 3.53 (3.03 - 4.03) | 6267 | 3.56 (3.07 - 4.05) | 1224 | 3.35 (2.85 - 3.9) | <0.001* |
| Reticulocyte Count, Automated | 1213 | 1.8 (1.2 - 2.7) | 992 | 1.8 (1.1 - 2.6) | 221 | 2.2 (1.3 - 3.2) | 0.002* |
| Sodium | 7513 | 138 (135 - 141) | 6282 | 138 (135 - 141) | 1231 | 137 (133 - 142) | 0.11 |
| Sodium, Whole Blood | 877 | 136 (131 - 141) | 551 | 137 (132 - 142) | 326 | 135 (131 - 140) | 0.001* |
| Thyroid Stimulating Hormone | 1344 | 2.1 (1.1 - 4.1) | 1084 | 2 (1 - 4) | 260 | 2.25 (1.1 - 4.5) | 0.263 |
| Transferrin | 1478 | 141 (112 - 175) | 1270 | 146 (117 - 179) | 208 | 115 (83 - 147.25) | <0.001* |
| Troponin T | 2135 | 0.08 (0.04 - 0.22) | 1483 | 0.07 (0.03 - 0.19) | 652 | 0.11 (0.05 - 0.302) | <0.001* |
| Urea Nitrogen | 7511 | 24 (15 - 40) | 6280 | 22 (14 - 36) | 1231 | 37 (24 - 58) | <0.001* |
| Vitamin B12 | 740 | 862 (584.5 - 1330.5) | 649 | 837 (565 - 1269) | 91 | 1171 (700 - 1573) | 0.001* |
| White Blood Cells | 7478 | 12.4 (8.2 - 17.8) | 6255 | 12.3 (8.2 - 17.3) | 1223 | 14 (7.9 - 20.5) | <0.001* |

* - significant *P*-values.

**Supplementary Table 3.**Results of the univariate logistic regression of all laboratory parameters

| **Laboratory parameter** | ***P-value*** | **aOR (95% CI)** |
| --- | --- | --- |
| decreased Red Cell Distribution Width - Standard Deviation (RDW-SD) | 0.007* | 10.31 (1.92 – 55.42) |
| increased Hemoglobin | <0.001* | 6.95 (2.61 – 18.5) |
| increased Phosphate | <0.001* | 4.97 (4.29 – 5.77) |
| increased Red Blood Cells | <0.001* | 4.95 (2.42 – 10.1) |
| increased Red Cell Distribution Width - Standard Deviation (RDW-SD) | <0.001* | 4.66 (3.38 – 6.44) |
| increased Hematocrit | <0.001* | 4.65 (2.67 – 8.1) |
| increased Calcium, Total | <0.001* | 4.19 (2.5 – 7.01) |
| increased Lactate Dehydrogenase (LDH) | <0.001* | 3.73 (3.16 – 4.4) |
| increased Anion Gap | <0.001* | 3.67 (3.17 – 4.24) |
| increased Urea Nitrogen | <0.001* | 3.59 (3.06 – 4.22) |
| decreased Fibrinogen, Functional | <0.001* | 3.25 (2.38 – 4.45) |
| increased Red Cell Distribution Width (RDW) | <0.001* | 3.09 (2.72 – 3.51) |
| increased Magnesium | <0.001* | 3.07 (2.38 – 3.97) |
| increased Bilirubin, Direct | <0.001* | 3.04 (1.74 – 5.32) |
| decreased Iron Binding Capacity, Total | 0.001* | 3.03 (1.62 – 5.67) |
| decreased Transferrin | 0.001* | 3.03 (1.62 – 5.67) |
| decreased Glucose | <0.001* | 2.98 (2.24 – 3.97) |
| increased Partial Thromboplastin Time (PTT) | <0.001* | 2.92 (2.55 – 3.34) |
| increased Potassium | <0.001* | 2.76 (2.31 – 3.29) |
| decreased pH | <0.001* | 2.69 (2.32 – 3.12) |
| increased N-terminal proBrain Natriuretic Peptide (NT-proBNP) | 0.016* | 2.67 (1.2 – 5.93) |
| decreased Glucose | 0.002* | 2.55 (1.42 – 4.61) |
| increased Creatinine | <0.001* | 2.53 (2.21 – 2.88) |
| increased Lactate | <0.001* | 2.5 (2.19 – 2.85) |
| decreased White Blood Cells | <0.001* | 2.49 (1.98 – 3.13) |
| decreased Neutrophils | <0.001* | 2.48 (1.79 – 3.42) |
| increased Ferritin | <0.001* | 2.44 (1.67 – 3.56) |
| increased Bilirubin, Total | <0.001* | 2.39 (2.06 – 2.76) |
| decreased Bicarbonate | <0.001* | 2.39 (2.1 – 2.71) |
| decreased Absolute Basophil Count | <0.001* | 2.37 (1.89 – 2.97) |
| increased International Normalized Ratio / Prothrombin Time (INR/PT) | <0.001* | 2.31 (1.88 – 2.84) |
| increased Prothrombin Time (PT) | <0.001* | 2.27 (1.86 – 2.78) |
| increased Vitamin B12 | 0.001* | 2.24 (1.41 – 3.57) |
| increased Mean Corpuscular Volume (MCV) | <0.001* | 2.23 (1.94 – 2.58) |
| increased Free Calcium | 0.002* | 2.21 (1.33 – 3.69) |
| decreased Albumin | <0.001* | 2.17 (1.72 – 2.74) |
| decreased Calculated Total CO_2_ | <0.001* | 2.16 (1.86 – 2.5) |
| increased Sodium | <0.001* | 2.12 (1.71 – 2.64) |
| increased Creatine Kinase, MB Isoenzyme | <0.001* | 2.11 (1.72 – 2.61) |
| decreased Chloride | <0.001* | 2.1 (1.77 – 2.5) |
| increased Bicarbonate | 0.002* | 2.08 (1.32 – 3.27) |
| decreased Absolute Neutrophil Count | 0.004* | 2.08 (1.27 – 3.43) |
| increased Aspartate Aminotransferase | <0.001* | 2.06 (1.79 – 2.36) |
| decreased Thyroid Stimulating Hormone | 0.01* | 2.06 (1.19 – 3.58) |
| increased Absolute Eosinophil Count | 0.023* | 2.01 (1.1 – 3.69) |
| decreased Sodium | <0.001* | 1.97 (1.68 – 2.3) |
| increased Cortisol | <0.001* | 1.96 (1.44 – 2.67) |
| increased Lymphocytes | 0.011* | 1.83 (1.15 – 2.9) |
| increased Metamyelocytes | <0.001* | 1.73 (1.45 – 2.08) |
| decreased Mean Cellular Haemoglobin Concentration (MCHC) | <0.001* | 1.72 (1.51 – 1.97) |
| increased Absolute Neutrophil Count | 0.001* | 1.7 (1.24 – 2.32) |
| increased Mean Cellular Haemoglobin (MCH) | <0.001* | 1.69 (1.46 – 1.96) |
| decreased Platelet Count | <0.001* | 1.68 (1.48 – 1.91) |
| increased Troponin T | 0.028* | 1.63 (1.05 – 2.52) |
| increased Reticulocyte Count, Automated | 0.005* | 1.57 (1.15 – 2.15) |
| decreased Calcium, Total | <0.001* | 1.57 (1.37 – 1.79) |
| increased Myelocytes | <0.001* | 1.55 (1.26 – 1.91) |
| increased pCO_2_ | <0.001* | 1.53 (1.3 – 1.8) |
| increased Chloride | <0.001* | 1.53 (1.32 – 1.78) |
| decreased Free Calcium | <0.001* | 1.53 (1.28 – 1.84) |
| decreased Absolute Monocyte Count | 0.027* | 1.44 (1.04 – 2) |
| increased Creatine Kinase | <0.001* | 1.43 (1.18 – 1.75) |
| decreased Sodium, Whole Blood | 0.018* | 1.43 (1.06 – 1.93) |
| decreased Absolute Eosinophil Count | 0.003* | 1.43 (1.13 – 1.81) |
| increased Alkaline Phosphatase | <0.001* | 1.41 (1.23 – 1.62) |
| increased Potassium, Whole Blood | 0.014* | 1.4 (1.07 – 1.84) |
| increased Lipase | 0.009* | 1.4 (1.09 – 1.81) |
| increased Alanine Aminotransferase | <0.001* | 1.39 (1.22 – 1.6) |
| increased Bands | 0.001* | 1.39 (1.15 – 1.67) |
| increased White Blood Cells | <0.001* | 1.39 (1.2 – 1.6) |
| decreased pCO_2_ | 0.006* | 1.27 (1.07 – 1.51) |
| decreased Red Blood Cells | 0.019* | 1.26 (1.04 – 1.53) |
| decreased Monocytes | 0.009* | 1.24 (1.05 – 1.46) |
| decreased MCH | 0.038* | 1.23 (1.01 – 1.5) |
| decreased Hemoglobin | 0.042* | 1.21 (1.01 – 1.45) |
| decreased pO_2_ | 0.033* | 0.79 (0.64 – 0.98) |
| decreased Phosphate | 0.005* | 0.78 (0.65 – 0.93) |
| increased Glucose | 0.04* | 0.73 (0.55 – 0.99) |
| decreased Eosinophils | <0.001* | 0.72 (0.61 – 0.84) |
| increased Haptoglobin | <0.001* | 0.59 (0.46 – 0.77) |
| decreased Iron | <0.001* | 0.53 (0.39 – 0.71) |
| increased Fibrinogen, Functional | <0.001* | 0.48 (0.39 – 0.61) |

* - significant *P*-values.

**Supplement Table 4.** e-SEPSS scoring model for predicting mortality.

| **Variable** | **Cut-off values** | **e-SEPSS points** |
| --- | --- | --- |
| Chlorides | <96 mEq/L | 1 |
| MCH | >32 pg | 1 |
| RDW | >15.5% | 1 |
| Phosphates | >4.5 mg/dL | 1 |
| PTT | >35 or >36.5 sec | 1 |
| LDH | >250 IU/L | 1 |
| pH | <7.35 units | 1 |
| SUM: | | /7 |

**Supplementary Table 5.** In-hospital mortality stratified according to the number of points calculated with e-SEPSS

| **Number of e-SEPSS points** | **Validation group (N=6542)**  **% (N)** | **Development group (N=1004)**  **% (N)** | ***P-value*** |
| --- | --- | --- | --- |
| 0 | 2.7 (1735) | 4.1 (74) | 0.489 |
| 1 | 6.6 (1936) | 12.8 (187) | 0.001* |
| 2 | 15.3 (1336) | 17.1 (269) | 0.463 |
| 3 | 30.8 (878) | 30.4 (227) | 0.963 |
| 4 | 44.9 (428) | 38.8 (152) | 0.216 |
| 5 | 60.1 (168) | 53.7 (67) | 0.383 |
| 6 | 78.4 (51) | 76.2 (21) | 1.000 |
| 7 | 90 (10) | 100 (7) | 1.000 |

* - significant *P*-values.

**Supplement Table 6.** General differences between model development (MD) and model validation (MV) group.

|  | | **Validation group (N=6542)** | **Development group (N=1004)** |
| --- | --- | --- | --- |
| Age | | 70 (57 - 82) | 69 (58 - 80) |
| Gender | Male | 3324 (50.8%) | 552 (55.0%) |
|  | Female | 3218 (49.2%) | 452 (45.0%) |
| Ethnicity | White | 4496 (68.7%) | 671 (66.8%) |
|  | Black / African American | 812 (12.4%) | 139 (13.8%) |
|  | Asian | 248 (3.8%) | 53 (5.3%) |
|  | Hispanic / Latino | 271 (4.1%) | 32 (3.2%) |
|  | Other / Unable to obtain | 715 (10.9%) | 109 (10.9%) |
| Hospitalization  outcome | Alive | 5551 (84.9%) | 744 (74.1%) |
|  | Dead | 991 (15.1%) | 260 (25.9%) |
| Hospitalization duration | | 6 (4 - 9) | 11 (7 - 18) |
| **Comorbidities** | | | |
| Hypertension | | 4066 (62.2%) | 632 (62.9%) |
| Cerebrovascular disorders | | 394 (6.0%) | 75 (7.5%) |
| Diabetes mellitus | | 2181 (33.3%) | 361 (36.0%) |
| Neoplasms | | 1551 (23.7%) | 282 (28.1%) |
| Ischemic heart disease | | 1518 (23.2%) | 241 (24.0%) |
| Diseases of pulmonary circulation | | 373 (5.7%) | 95 (9.5%) |
| Heart failure | | 1793 (27.4%) | 352 (35.1%) |
| Chronic rheumatic heart disease | | 155 (2.4%) | 55 (5.5%) |
| Liver disease | | 805 (12.3%) | 224 (22.3%) |
| Asthma | | 586 (9.0%) | 80 (8.0%) |
| Chronic Obstructive Pulmonary Disease | | 981 (15.0%) | 172 (17.1%) |
| Renal failure | | 1637 (25.0%) | 291 (29.0%) |
| Hereditary and degenerative diseases of the CNS | | 337 (5.2%) | 27 (2.7%) |
| **Laboratory parameters** | | | |
| Base Excess | | -2 (-6 - 0) | -2 (-6 - 0) |
| Calculated Total CO_2_ | | 23 (19 - 27) | 23 (20 - 27) |
| Free Calcium | | 1 (1 - 1) | 1 (1 - 1) |
| Glucose | | 132 (103 - 183) | 145 (109 - 193) |
| Lactate | | 1.9 (1.3 - 2.8) | 2.1 (1.5 - 3.2) |
| Oxygen | | 50 (40 - 100) | 50 (40 - 80) |
| Oxygen Saturation | | 84 (69 - 95) | 77 (66 - 93) |
| pCO_2_ | | 40 (33 - 48) | 40 (34 - 47) |
| pH | | 7.36 (7.28 - 7.41) | 7.36 (7.29 - 7.41) |
| pO_2_ | | 69 (44 - 106) | 53 (39 - 87.5) |
| Potassium, Whole Blood | | 4.1 (3.6 - 4.9) | 4.1 (3.5 - 4.9) |
| Sodium, Whole Blood | | 136 (132 - 141) | 137 (131 - 142) |
| Alanine Aminotransferase (ALT) | | 29 (16 - 63) | 29 (16 - 73) |
| Albumin | | 2.9 (2.5 - 3.3) | 2.8 (2.4 - 3.2) |
| Alkaline Phosphatase | | 96 (69 - 153) | 102.5 (70 - 160.5) |
| Anion Gap | | 15 (13 - 18) | 17 (14 - 20) |
| Asparate Aminotransferase (AST) | | 37 (22 - 81) | 45 (25 - 105) |
| Bicarbonate | | 22 (19 - 25) | 21 (18 - 24) |
| Bilirubin, Direct | | 2 (1 - 4) | 2 (1 - 4) |
| Bilirubin, Indirect | | 1 (0 - 2) | 1 (0 - 2) |
| Bilirubin, Total | | 0.6 (0.4 - 1.4) | 0.8 (0.4 - 2) |
| C-Reactive Protein | | 110 (53 - 183) | 109 (52 - 174) |
| Calcium, Total | | 8.2 (7.6 - 8.7) | 8.1 (7.6 - 8.7) |
| Chloride | | 103 (99 - 107) | 101 (97 - 106) |
| Cortisol | | 23 (15 - 37) | 17 (12 - 26) |
| Creatine Kinase (CK) | | 99 (42 - 289) | 126 (44 - 457) |
| Creatine Kinase, MB Isoenzyme | | 4 (2 - 8) | 4 (2 - 10) |
| Creatinine | | 1.1 (0.8 - 1.8) | 1.4 (0.9 - 2.4) |
| Ferritin | | 402 (186 - 854) | 612 (276 - 1247) |
| Glucose | | 119 (97 - 158) | 130 (101 - 180.5) |
| Haptoglobin | | 218 (130 - 306) | 211 (117 - 299) |
| Iron | | 29 (16 - 50) | 33 (20 - 59) |
| Iron Binding Capacity, Total | | 190 (151 - 231) | 169 (135 - 214) |
| Lactate Dehydrogenase (LD) | | 243 (185 - 342) | 284 (213 - 420) |
| Lipase | | 26 (15 - 56) | 31.5 (15 - 75) |
| Magnesium | | 1.9 (1.6 - 2.1) | 1.9 (1.6 - 2.2) |
| NTproBNP | | 4108 (1229 - 10933) | 4420 (1092 - 13154) |
| Phosphate | | 3.2 (2.5 - 4) | 3.6 (2.8 - 4.7) |
| Potassium | | 4 (3.6 - 4.5) | 4.2 (3.7 - 4.7) |
| Sodium | | 138 (135 - 141) | 137 (134 - 141) |
| Thyroid Stimulating Hormone | | 2 (1 - 4) | 2 (1 - 4) |
| Transferrin | | 146 (116 - 178) | 130 (104 - 165) |
| Troponin T | | 0 (0 - 0) | 0 (0 - 0) |
| Urea Nitrogen | | 23 (14 - 39) | 29 (18 - 49) |
| Vitamin B12 | | 841 (565 - 1352) | 927 (633 - 1253) |
| Absolute Lymphocyte Count | | 1 (1 - 1) | 1 (0 - 1) |
| Atypical Lymphocytes | | 0 (0 - 0) | 0 (0 - 0) |
| Bands | | 2 (0 - 8) | 2 (0 - 6) |
| Basophils | | 0.2 (0 - 0.3) | 0.1 (0 - 0.3) |
| Eosinophils | | 0.3 (0 - 1.1) | 0 (0 - 1) |
| Fibrinogen, Functional | | 428 (262 - 587) | 397 (214 - 594) |
| Hematocrit | | 32.3 (28.2 - 36.4) | 31.4 (26.6 - 36.2) |
| Hemoglobin | | 10.5 (9.1 - 11.9) | 10.1 (8.5 - 11.7) |
| INR(PT) | | 1 (1 - 2) | 1 (1 - 2) |
| Lymphocytes | | 8.05 (4.5 - 13.9) | 6.8 (3.4 - 12.7) |
| MCH | | 29.9 (28.2 - 31.5) | 29.7 (28 - 31.5) |
| MCHC | | 32.5 (31.4 - 33.6) | 32.2 (30.9 - 33.3) |
| MCV | | 92 (87 - 96) | 92.5 (87 - 98) |
| Metamyelocytes | | 0 (0 - 1) | 0 (0 - 1) |
| Monocytes | | 5 (3 - 7.5) | 5.95 (3 - 8.6) |
| Myelocytes | | 0 (0 - 0) | 0 (0 - 1) |
| Neutrophils | | 82.15 (73 - 88.3) | 82.15 (73 - 88) |
| Platelet Count | | 190 (131 - 265) | 171 (103 - 249) |
| PT | | 15 (13 - 19) | 16 (13 - 20) |
| PTT | | 32 (29 - 39) | 32 (28 - 39) |
| RDW | | 14.8 (13.7 - 16.6) | 15.5 (14.2 - 17.65) |
| Red Blood Cells | | 3.54 (3.06 - 4.03) | 3.43 (2.87 - 4) |
| Reticulocyte Count, Automated | | 2 (1 - 3) | 2 (1 - 3) |
| White Blood Cells | | 12.3 (8.1 - 17.5) | 13.5 (8.7 - 20.4) |
| Absolute Basophil Count | | 0 (0 - 0) | 0 (0 - 0) |
| Absolute Eosinophil Count | | 0 (0 - 0) | 0 (0 - 0) |
| Absolute Monocyte Count | | 1 (0 - 1) | 1 (0 - 1) |
| Absolute Neutrophil Count | | 10 (6 - 15) | 11 (6 - 17) |
| Immature Granulocytes | | 1 (1 - 1) | 1 (1 - 1) |
| RDW-SD | | 49 (45 - 55) | 52 (47 - 59) |


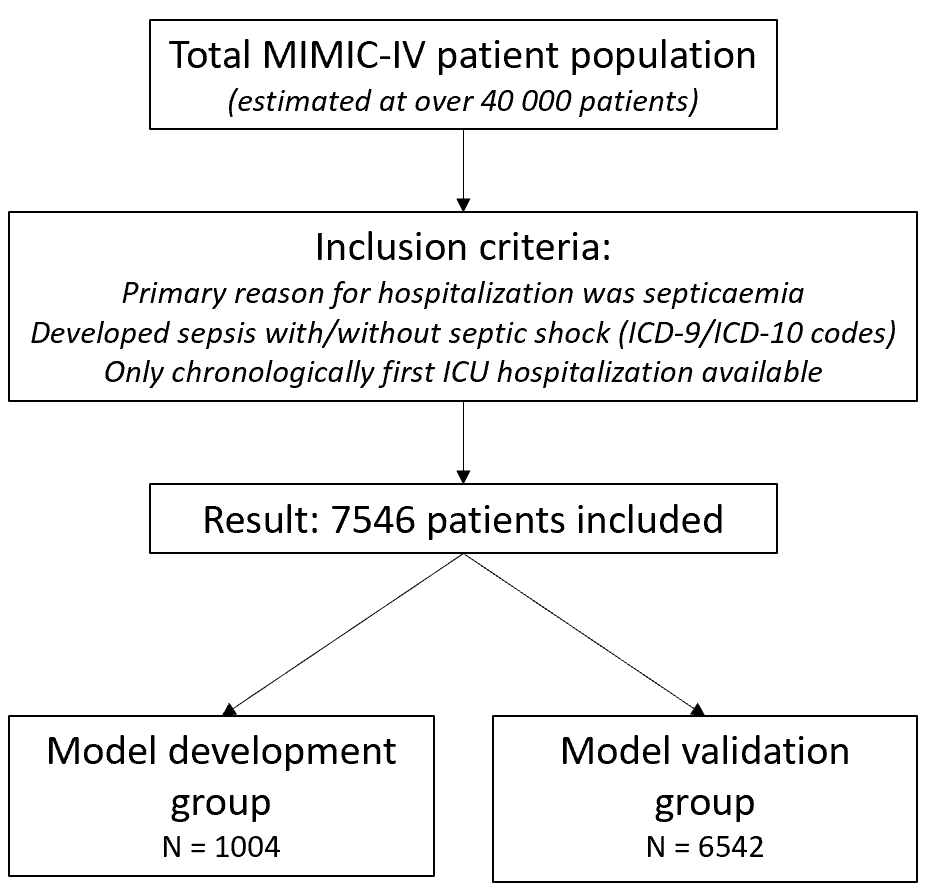


**Supplementary Figure 1.** Flowchart describing the patient selection process and inclusion criteria for the study. Details about the model validation and model development group can be found in the Methods section.


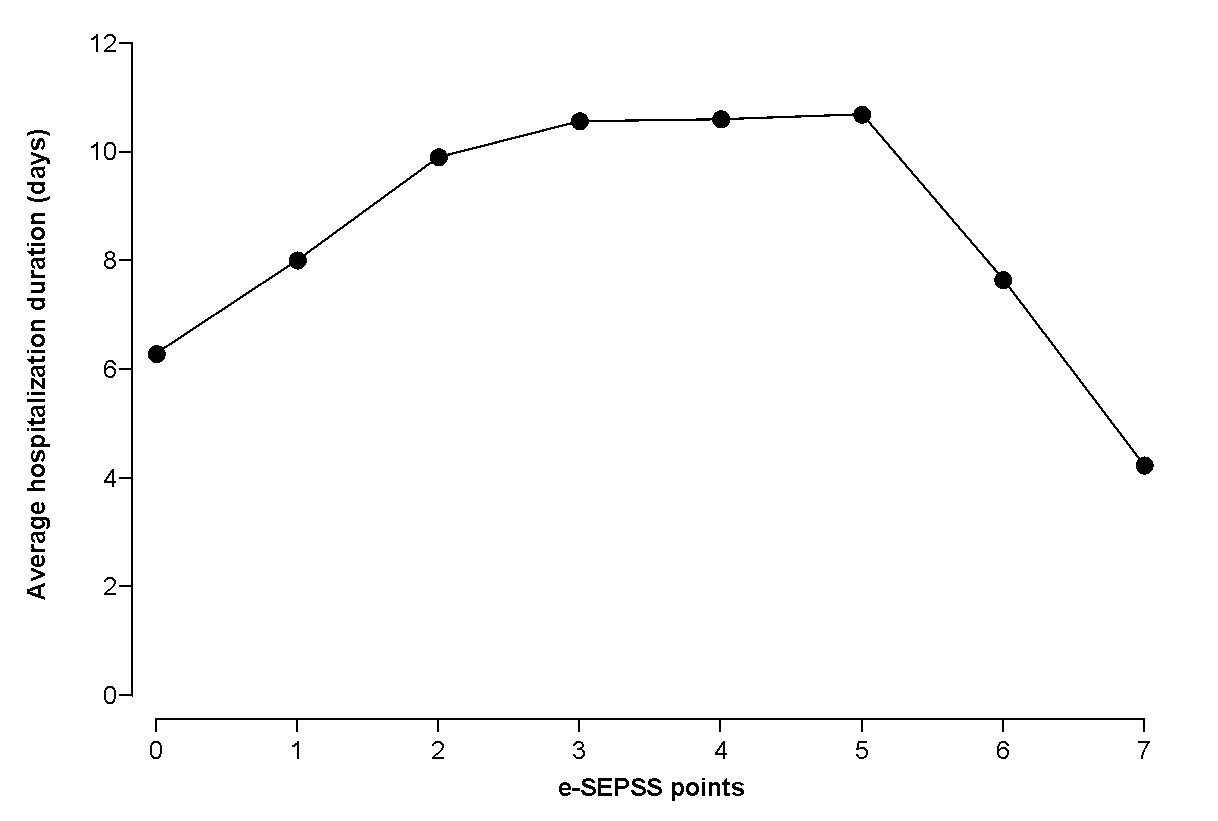
**Supplementary Figure 2.** Kaplan-Meier survival curves for groups with different number of e-SEPSS points in the first 50 days of hospitalization.

**
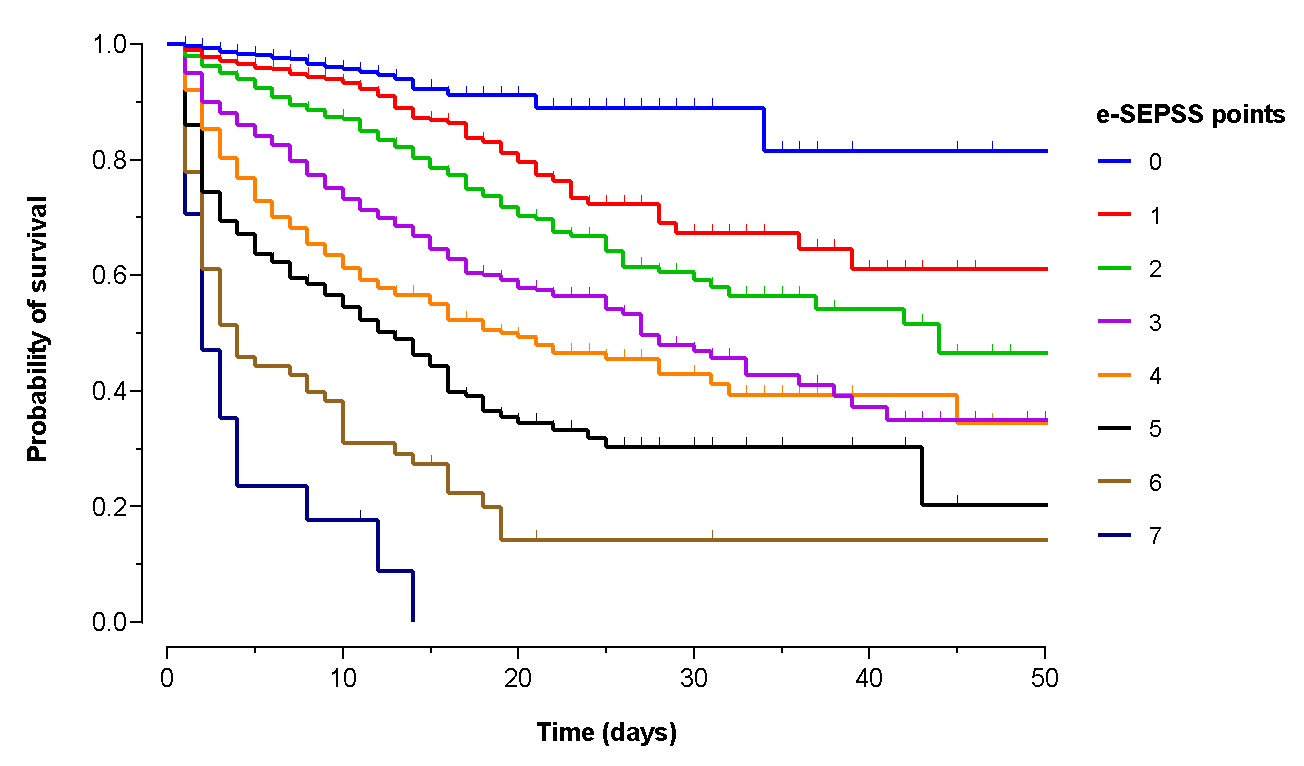
**

**Supplementary Figure 3.** Average hospital duration according to the number of e-SEPSS points.
